# Deep sequencing on genome-wide scale reveals the unique composition and expression patterns of microRNAs in developing pollen of *Oryza sativa*

**DOI:** 10.1186/gb-2011-12-6-r53

**Published:** 2011-06-16

**Authors:** Li Qin Wei, Long Feng Yan, Tai Wang

**Affiliations:** 1 Research Center for Molecular and Developmental Biology, Key Laboratory of Photosynthesis and Environmental Molecular Physiology, Institute of Botany, Chinese Academy of Sciences, 20 Nanxincun, Xiangshan, Beijing 100093, China; 2 National Center for Plant Gene Research, 20 Nanxincun Xiangshan Haidianqu, Beijing 100093, China; 3 Graduate School of Chinese Academy of Sciences, 19A Yuquanlu Shijingshanqu, Beijing 100049, China

## Abstract

**Background:**

Pollen development in flowering plants requires strict control of the gene expression program and genetic information stability by mechanisms possibly including the miRNA pathway. However, our understanding of the miRNA pathway in pollen development remains limited, and the dynamic profile of miRNAs in developing pollen is unknown.

**Results:**

Using next-generation sequencing technology, we pyrosequenced small RNA populations from rice uninucleate microspores to tricellular pollen and control sporophytic tissues at the genome-wide level. We identified 292 known miRNAs, including members of all 20 families conserved in plants, and 75 novel miRNAs. Of the 292 known miRNAs, 202 were expressed, with 103 enriched, in developing pollen. More than half of these novel miRNAs displayed pollen-or stage-specific expression. Furthermore, analyzing the 367 miRNAs and their predicted targets indicated that correlation in expression profiles of pollen-enriched known miRNAs and their targets significantly differs from that of sporophyte-enriched known miRNAs and their targets in some functional terms, while novel miRNAs appeared to negatively regulate their targets. Importantly, gene ontology abundance analysis demonstrated chromatin assembly and disassembly was important in the targets of bicellular pollen-expressed novel miRNAs. Principal component analysis revealed pollen of all three stages was discriminated from sporophytes, largely because of the novel and non-conserved known miRNAs.

**Conclusions:**

Our study, for the first time, revealed the differences in composition and expression profiles of miRNAs between developing pollen and sporophytes, with novel and non-conserved known miRNAs the main contributors. Our results suggest the important roles of the miRNA pathway in pollen development.

## Background

MicroRNAs (miRNAs) and small interfering RNAs (siRNAs) are two types of small non-coding RNAs (20 to 24 nucleotides in length) identified in nearly all eukaryotes. The pool of small RNAs in plants is highly complex, consisting primarily of many low-abundant siRNAs and a small number of highly expressed 21-nucleotide sequences; most of the latter are miRNAs [1,2]. Most miRNA loci are encoded by independent transcriptional units in intergenic regions that are transcribed by RNA polymerase II. In plants, miRNAs are processed from stem-loop regions of long primary transcripts by a Dicer-like enzyme and are loaded into silencing complexes, where they generally direct cleavage of complementary mRNAs. Although miRNAs were identified in plants just recently, studies have revealed that miRNAs play crucial roles in each major stage of plant development, often targeting the transcription factors that mediate transition from one developmental stage to the next [3].

Haploid pollen (also called the gametophyte) is a key regulator of sexual reproduction in flowering plants and is produced from diploid pollen mother cells via meiosis. In contrast to animals, in which products of meiosis directly develop into sperm cells, in plants, the product of meiosis undergoes a unique postmeiotic pollen development process, finally giving rise to sperm cells. During this process, haploid uninucleate microspores (UNMs) generated from meiosis first undergo asymmetric mitosis to generate bicellular pollen (BCP) consisting of a large vegetative cell and a small generative cell enclosed in the vegetative cell. The two types of cells have different fates: the vegetative cell exits the cell cycle and can develop into a polarly growing pollen tube, whereas the generative cell undergoes further mitosis to produce two sperm cells. This postmeiotic pollen development is orderly and precisely regulated [4]; however, the mechanism underlying the main developmental events remains largely unknown.

Recent transcriptomics studies have revealed that the number of genes expressed in pollen greatly decreases from the UNM stage to the tricellular pollen (TCP) stage, whereas stage-specific transcripts showed a 'U-type' change, with the lowest number at the BCP stage in *Oryza sativa *and *Arabidopsis thaliana *[5]. These data suggest that fine-tuned gene expression and function are essential to guarantee pollen development. The miRNA pathways in pollen are of interest because previous transcriptomic analysis showed key transcripts involved in miRNA pathway, such as those encoding Argonaute (AGO)1, 2, 4 and 7, Dicer-like protein (DCL)1 to 3 and RNA-dependent RNA polymerase 1, 2 and 6, were turned off during pollen development [6]. However, recent research demonstrated that several miRNA pathway genes were expressed and some enriched in pollen grains of *Arabidopsis *[7-10]. Many miRNAs known to function in somatic development have been identified in *Arabidopsis *mature pollen [9,11]. Using 454 sequencing, Grant-Downton *et al*. [10] revealed diverse small RNAs in mature pollen of *Arabidopsis*, some of which were possibly specific to mature pollen. These studies indicate the existence of an miRNA pathway in pollen. However, mature pollen is terminally differentiated and at a developmental 'standstill', so the present studies could not define the importance of miRNAs in pollen development. Sequencing small RNAs from developing pollen of different stages is necessary to obtain a global picture of the temporal dynamics of small RNA diversity [10]. Therefore, genome-wide knowledge about the composition of miRNAs and dynamic changes in the miRNAs during pollen development is important to understand the mechanism of fine-tuned pollen development.

Rice (*O. sativa*) is one of the most important cereal crops; it feeds half of the world's population and has been used as an excellent model system for studying monocots after *Arabidopsis*. Besides the importance of rice, knowledge of the molecular mechanisms underlying rice pollen development is essential to manipulate male fertility for heterosis utilization. In this study, we used Solexa high-throughout sequencing technology to sequence the small RNA population from developing rice pollen at the UNM, BCP and TCP stages, with sporophytic tissues-roots, leaves, and callus cells-used as controls. We obtained millions of high-quality readouts from each sample. Further analyses identified 292 known miRNAs, including members of all 20 families conserved in plants, and 75 novel miRNAs. Most of the known miRNAs (hereafter called kn-miRs) were expressed in developing pollen, and most of the predicted novel miRNAs (nov-miRs) were pollen specific. We also predicted 1,353 genes possibly targeted by the 367 identified miRNAs and correlated the expression profiles of miRNAs and their targets. We supply novel insights into the dynamic profiles of miRNAs in developing pollen.

## Results

### Sequencing and data analysis

To investigate the miRNA component of small RNAs and the dynamic changes of the miRNAs during pollen development, we purified the cells of UNMs, BCP and TCP from rice and sequenced their small RNAs using Solexa high-throughput technology. As a control, small RNAs of three sporophytic tissues (one-month-old callus and two-week-old roots and leaves) were sequenced simultaneously. Sequencing of the UNM, BCP and TCP libraries generated 6,450,464, 13,497,446 and 14,952,272 raw readouts, respectively. After removing sequences of low quality, adaptor contaminants and RNAs smaller than 18 nucleotides, we obtained 5,513,740, 10,256,852, and 12,212,973 high-quality 18-to 30-nucleotide small RNAs from UNMs, BCP and TCP, respectively, 10,884,533 from callus cells, 10,777,375 from leaves and 10,424,066 from roots (Table 1). These high-quality small RNAs were used for further analysis.

**Table 1:** Statistics of small RNA sequences from the individual libraries

	**UNM (%)**	**BCP (%)**	**TCP (%)**	**Callus (%)**	**Leaf (%)**	**Root (%)**
	
Total reads	6,450,464 (100)	13,497,446 (100)	14,952,272 (100)	14,009,265 (100)	13,636,372 (100)	12,663,429 (100)
High quality reads^a^	5,982,567 (92.75)	12,037,307 (89.18)	13,573,834 (90.78)	12,447,058 (88.85)	12,323,660 (90.37)	11,328,710 (89.46)
Sequences of 18 to 30 nucleotides^a^	5,513,740 (85.48)	10,256,852 (75.99)	12,212,973 (81.68)	10,884,533 (77.70)	10,777,375 (79.03)	10,424,066 (82.32)
Sequences matched to the genome^b^	4,415,708 (80.09)	8,857,467 (86.36)	10,855,875 (88.89)	9,007,545 (82.76)	9,576,588 (88.86)	8,563,670 (82.15)
rRNA etc.^b^	3,302,769 (59.90)	6,424,472 (62.64)	10,721,753 (87.79)	6,752,856 (62.04)	8,556,637 (79.39)	7,154,132 (68.63)
Exon_antisense^b^	31,248 (0.57)	56,456 (0.55)	15,122 (0.12)	42,744 (0.39)	22,426 (0.21)	18,385 (0.18)
Exon_sense^b^	317,700 (5.76)	343,714 (3.35)	374,377 (3.07)	144,997 (1.33)	294,639 (2.73)	164,069 (1.57)
Intron_antisense^b^	12,444 (0.23)	34,657 (0.34)	5,858 (0.05)	34,376 (0.32)	10,330 (0.10)	16,897 (0.16)
Intron_sense^b^	24,045 (0.44)	13,997 (0.14)	13,105 (0.11)	42,672 (0.39)	31,089 (0.29)	26,438 (0.25)
Kn-miRs^b^	34,930 (0.63)	73,567 (0.72)	23,574 (0.19)	920,584 (8.46)	438,823 (4.07)	383,694 (3.68)
Repeat-associated small RNAs^b^	651,296 (11.81)	1,665,020 (16.23)	177,549 (1.45)	1,113,638 (10.23)	428,592 (3.98)	912,267 (8.75)
Un-annotated small RNAs^b^	1,139,308 (20.66)	1,644,969 (16.04)	881,635 (7.22)	1,832,666 (16.84)	994,839 (9.23)	1,748,184 (16.77)

^a^The percentage to the total reads. ^b^The percentage to the sequences of 18 to 30 nucleotides.

**Figure 1:**
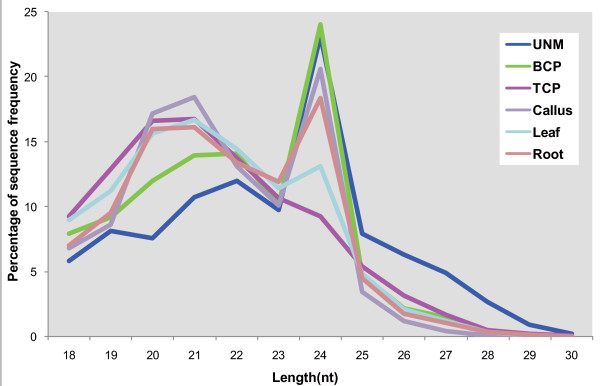
**Size distribution of small RNAs in the different libraries**. Nt, nucleotides.

Of the millions of high-quality small RNAs from the individual libraries, 72.4% were 20 to 24 nucleotides in length, which is the typical size range for Dicer-derived products [12]. The major component of small RNAs in UNMs was 24 nucleotides long. Throughout pollen development, the proportion of 24-nucleotide small RNAs decreased and the 21-nucleotide population increased in BCP, while TCP contained mostly 21-nucleotide small RNAs (Figure 1). In the sporophytic samples, although two peaks occurred in all three libraries, 24-nucleotide small RNAs were the most abundant in callus cells and roots, and 21-nucleotide small RNAs were the most abundant in leaves (Figure 1).

In general, the small RNA library generated by sequencing was complex in composition. Besides the miRNAs and siRNAs, the library includes large numbers of degradation fragments derived from other coding and noncoding transcripts [13,14]. Therefore, to annotate small RNAs, we first mapped these small RNAs of 18 to 30 nucleotides to the rice genome using SOAP [15]. More than 80% of the small RNAs mapped perfectly to the genome (Table 1). Furthermore, we removed sequences corresponding to known non-coding RNAs (for example, rRNAs, tRNAs, small nuclear RNAs (snRNAs) and small nucleolar RNAs (snoRNAs); Table 1) and possibly degraded species of mRNAs. Finally, we obtained 1,825,534, 3,383,556 and 1,082,758 small RNAs from UNMs, BCP and TCP, respectively, and 3,866,888, 1,862,254 and 3,044,145 from callus cells, leaves and roots, respectively, which were candidates for identifying miRNAs (including the kn-miRs, repeat-associated small RNAs and un-annotated small RNAs in Table 1).

By mapping these candidates to their precursor sequences in the rice miRNA database available in miRBase [16-18], we identified 292 kn-miRs in pollen and/or sporophytic samples (Additional files 1 and 2); 202 of these kn-miRs were expressed in pollen samples, with 141 in UNMs, 152 in BCP and 143 in TCP; 281 were expressed in sporophytic tissues, with 210 in callus cells, 239 in roots and 223 in leaves. In addition, we detected 20 conserved kn-miR families in these libraries, and 16 of the families, apart from miR394, miR395, miR398 and miR408, were sequenced in developing rice pollen (Additional files 1 and 2). Thus, to a certain extent, our sequencing depth was sufficient to reflect the expression profiles of miRNAs during pollen development, and most of the conserved miRNAs were also expressed in the developing pollen.

Recent studies have revealed that some small RNAs derived from highly repeated elements bind with different AGO proteins and are involved in important biological processes, such as chromatin maintenance and transposon control [19,20]. We used RepeatMasker software to identify small RNAs positioned at repeat loci and annotated them as repeat-associated small RNAs (Table 1). The remaining un-annotated small RNAs were further used to predict nov-miRs.

### Prediction of nov-miRs

miRNAs are derived from hairpin-like precursors, originating from a single-stranded RNA transcript through sequential processing by Dicer or Dicer-like (DCL) proteins [21,22]. miRNA precursors have a characteristic fold-back structure, which is the primary criterion to annotate nov-miRs [23]. Therefore, we predicted nov-miRs as follows. First, by folding the flanking genome sequence of the above un-annotated small RNAs, followed by analysis of structural features, we excluded small RNAs that cannot form the characteristic fold-back structure. Second, recently evolved/evolving miRNAs have a single locus in the genome [24-26], so small RNAs with multiple loci in the rice genome were excluded. Third, to minimize noise, we also eliminated small RNAs of low abundance (with total number of reads fewer than five) and those originating from both strands, which would generate siRNA-like small RNAs. Fourth, all the remaining un-annotated small RNA sequences were subjected to 'MIREAP', which recovers most kn-miRs with only a few exceptions whose structures cannot satisfy the common features of an miRNA gene [27]. Finally, to distinguish miRNAs from miniature inverted repeat transposable elements (MITEs), we blasted the precursor and mature sequences of the small RNAs with characteristic fold-back structure against the *Oryza *Repeat Database [28], and the homologs of repetitive sequences were discarded. From the above analyses, our predicated nov-miRs satisfied the following criteria: precursors had a characteristic fold-back structure, contained no repetitive sequences, and matched the genome only once, and most were located in the intergenic region; lengths of mature miRNA ranged from 20 to 24 nucleotides, and the number of reads was greater than five; the mature sequences could be sequenced in two or more libraries, or the miRNA* sequence could be identified in at least one library; and the targets of predicted miRNAs could be predicted using an upgraded version of miRU [29].

In total, we obtained 75 predicted nov-miRs (Additional file 3). Of these, 30 were expressed in UNMs, 39 in BCP, and 18 in TCP, and only 14, 18 and 12 in callus cells, leaves and roots, respectively (Additional file 4). Compared with the increased number of kn-miRs expressed in sporophytic tissues, more nov-miRs were identified in developing rice pollen, so more miRNAs still remain to be revealed in gametophytes.

### Expression profiles of miRNAs during pollen development

**Figure 2:**
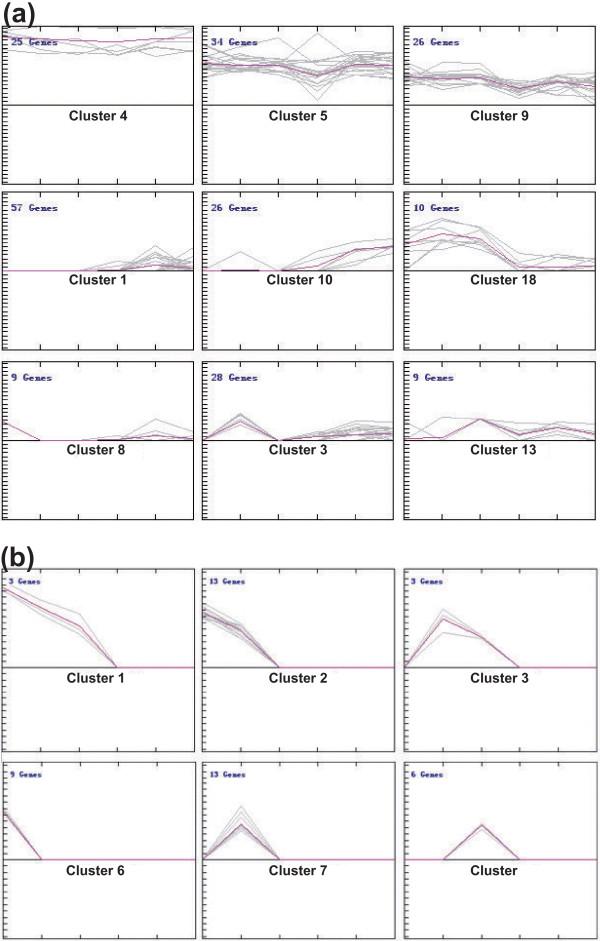
**Representive clusters of kn-miRs and nov-miRs by K-means support**. **(a) **Kn-miRs; **(b) **nov-miRs. The complete sets of clusters are available in Additional file 5 (kn-miRs) and Additional file 6 (nov-miRs). The six points from left to right in the x-axis represent UNMs, BCP, TCP, callus cells, root and leaf, respectively; the y-axis represents the log2 value of TPM.

To directly compare the expression patterns of these miRNAs in the developing pollen and in sporophytes, we normalized the counts to 1 million, and the abundance of each miRNA was expressed as transcripts per million (TPM). Using Z-score transformation [5], with ratio > 2.0 and Z-score > 2.0 cutoffs, we identified 103 kn-miRs expressed preferentially in developing pollen and 122 preferentially in sporophytic tissues (Additional file 1). Clustering analysis revealed a high proportion of kn-miRs expressed constitutively in all samples (clusters 4, 5 and 9 in Figure 2a) or preferentially in sporophytic tissues (clusters 1 and 10 in Figure 2a), and most of the conserved kn-miRs were in these clusters (Additional file 1). However, this analysis also showed some kn-miRs accumulated to a large extent in developing rice pollen or at individual stages of pollen development. For example, the members of clusters 8, 3 and 13 (Figure 2a) displayed UNM-, BCP-and TCP-enriched expression, respectively; and those in cluster 18 (Figure 2a) accumulated to a greater extent in pollen than in sporophytic tissues. Moreover, some conserved kn-miRs, such as osa-miR160e, osa-miR162b, osa-miR169e and n/o, osa-miR171a, osa-miR396a/b and osa-miR399h, were also present in pollen-enriched clusters (Additional file 1).

The expression features of these nov-miRs differ from those of kn-miRs (Additional files 5 and 6). More than half of the nov-miRs were expressed only in pollen libraries and some of them only in one pollen library (clusters 1 to 3 and 6 to 8 in Figure 2b). Nov-miRs in clusters 6, 7 and 8 (Figure 2b) displayed UNM-, BCP-and TCP-specific expression, respectively. These should represent a set of pollen-or stage-specific miRNAs. Therefore, in contrast to more kn-miRs being expressed constitutively or preferentially in sporophytic tissues, more nov-miRs were expressed preferentially and even specifically in pollen.

**Figure 3:**
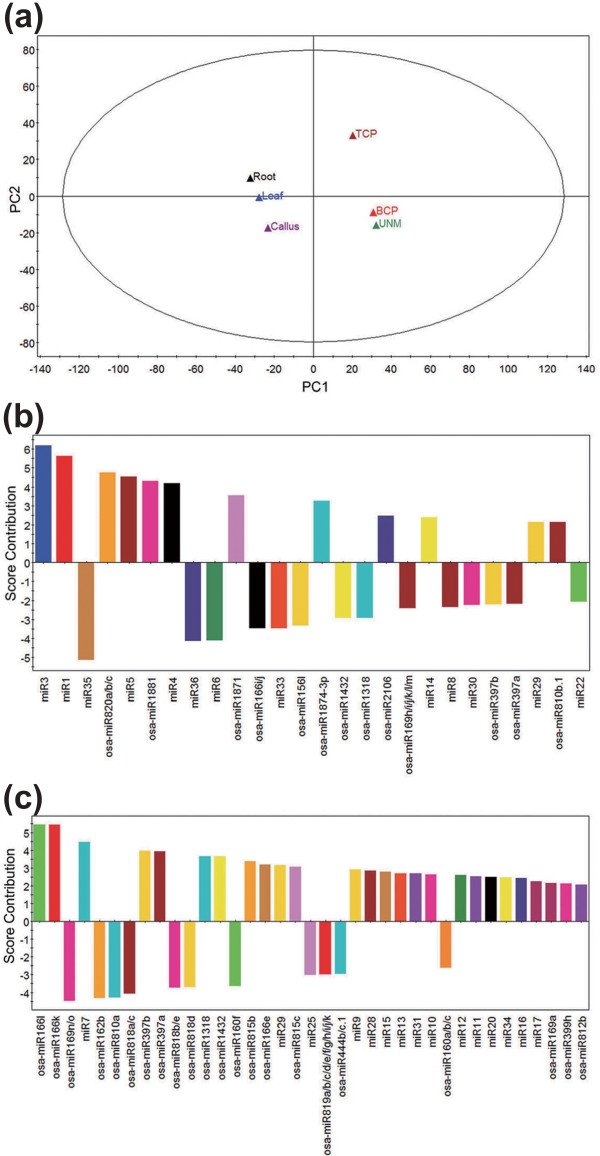
**Principal component analysis of all kn-miRs and nov-miRs from the six examined samples**. **(a) **Principal component analysis plot. The first (x-axis) and second principal component (y-axis) accounted for 44.9% and 17.2%, respectively, of the total variation in the data. **(b, c) **Score contributor plot for the first (b) and second (c) principal components. miRNAs with score contribution more than the absolute value of 2 are illustrated in the plot.

Furthermore, we compared the expression profiles of all miRNAs identified in pollen and sporophytic tissues using principal component analysis. The six samples were clustered into two groups (Figure 3a) that separated pollen from sporophytic tissues. As shown in the score contribution plot (Figure 3b), nov-miRs and non-conserved kn-miRs were the major contributors for differentiating the pollen from the sporophytes with regard to miRNA regulation. The miRNAs represented by the positive bars (Figure 3b; including miR3, miR1, miR5, miR4, miR14, miR29 and osa-miR820, osa-miR1881, osa-miR1871, osa-miR1874-3p, osa-miR2106, and osa-miR810b.1) contributed to pollen properties, while the negative-score miRNAs (Figure 3b; including miR35, miR36, miR6, miR8, miR30, miR22 and osa-miR166i/j, osa-miR156l, osa-miR1432, osa-miR1318, osa-miR169h-m, and osa-miR397a and b) contributed to sporophyte properties. Among the developing pollen samples, BCP was clustered with UNMs instead of TCP, which indicates that BCP is more similar to UNMs with regard to expression pattern of miRNAs. Also, miRNAs enriched in UNMs and BCP represented by the positive bars and miRNAs enriched in TCP represented by the negative bars (Figure 3c) might help to discriminate UNMs and BCP from TCP.

**Figure 4:**
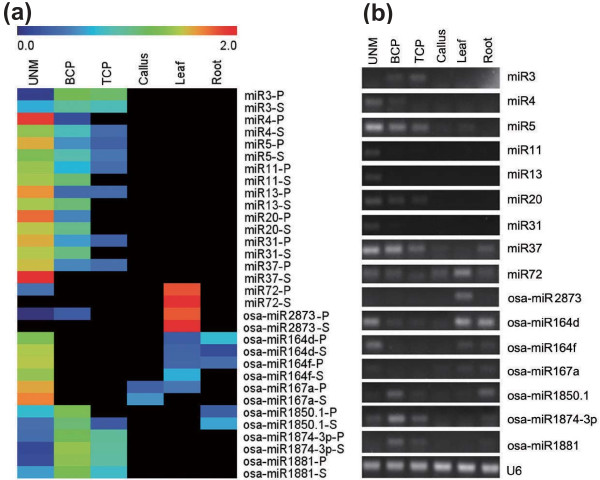
**Validation of expression profiles of miRNAs**. **(a) **Heatmap of stem-loop real-time RT-PCR and sequencing data. The bar represents the scale of the expression levels of miRNAs (log 2). P denotes expression detected by PCR and S that by Solexa sequencing. **(b) **Stem-loop semi-quantitative PCR of miRNAs with U6 as an internal control.

To validate the predicted nov-miRs and confirm the expression profile determined using Solexa sequencing, we performed stem-loop real-time quantification RT-PCR, a gold standard for accurate identification and quantification of miRNAs because of its high sensitivity, specificity and precision [29-31]. Because of the difficulty of cloning miRNAs with lower abundance, we selected nine predicted nov-miRs with relatively high expression and seven kn-miRs to validate. Of these, three were sequenced in only one library and the others in two or more libraries. The expression profiles of all were the same as those detected by Solexa sequencing (Figure 4; Additional file 7). This high confirmation rate indicates the reliability of our data and that our computational filters were strict enough for predicting nov-miRs.

### Target prediction and function analysis

Finding regulatory mRNA targets is essential for understanding the biological functions of miRNAs. We predicted targets of all identified miRNAs using the psRNATarget web service [29] and found 1,068 targets for 292 kn-miRs (Additional file 8a) and 285 targets for 75 nov-miRs (Additional file 8b). In total, we predicted 1,353 genes possibly targeted by the identified 367 miRNAs, with an average of 3.7 targets per miRNA, and each kn-miR (292 versus 1,068 targets) and nov-miR (75 versus 285) appeared to have similar numbers of targets on average.

The analysis of kn-miR targets showed that a high proportion of the targets were transcription factors (Additional file 8a). Besides transcription factor targets, targets involving defense response, hormone regulation and other metabolic pathways were overrepresented (Additional file 8a). This result echoes previous research on kn-miRs and their targets in sporophytic tissues [3,30]. Furthermore, Gene Ontology (GO) abundance analysis of targets of both pollen-and sporophyte-enriched kn-miRs revealed terms related to transcription regulation, transcription factor activity and metabolic regulation that were all statistically significant (false discovery rate *P *< 0.01; Additional file 9). However, hormone-signaling-related genes were overrepresented in pollen-enriched kn-miR targets, whereas lignin metabolism-related genes were overrepresented in sporophyte-enriched kn-miR targets (Additional file 9).

**Figure 5:**
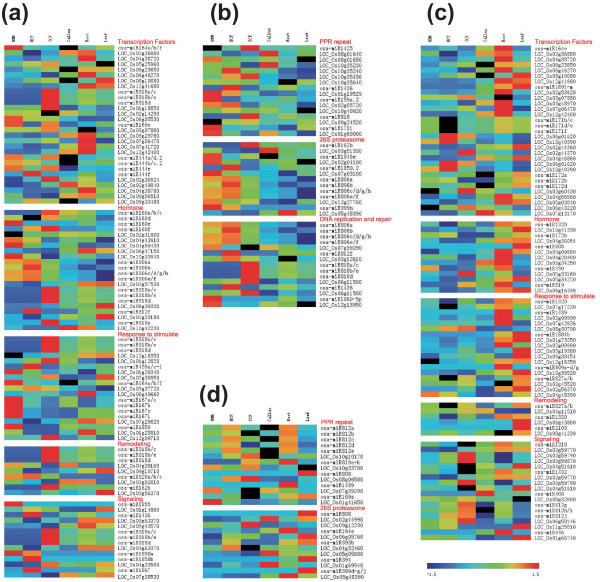
**Correlation of expression of miRNAs and their targets**. **(a) **Pollen-enriched miRNAs negatively associated with their targets. **(b) **A proportion of pollen-enriched miRNAs positively associated with their targets. **(c) **A proportion of sporophyte-enriched miRNAs positively associated with their targets. **(d) **Sporophyte-enriched miRNAs negatively associated with their targets. The bar represents the scale of relative expression levels of miRNAs and targets (log 2).

Furthermore, we compared the expression profiles of miRNAs and their targets using recently published transcriptome data for developing rice pollen [5] and found that the expression profiles of most of the kn-miRs were negatively correlated with those of their targets (Additional file 10a). In accordance with this result, the expression of most pollen-enriched kn-miRs was negatively related to that of their targets involved in transcription, hormone regulation, chromatin remodeling and defense response (Figure 5a), but the expression of about 30% of genes involved in these functions was positively related to their corresponding kn-miRs enriched in sporophytes (Figure 5c). Among targets involved in encoding pentatricopeptide repeat (PPR)-containing proteins and ubiquitin system members and in DNA repair, the expression of about 30% of them was positively correlated with that of corresponding pollen-enriched kn-miRs (Figure 5b), but the expression of almost all genes involved in these terms was negatively related to that of their sporophyte-enriched kn-miRs (Figure 5d). These data demonstrate the differential correlation of expression profiles of sporophyte-enriched and pollen-enriched kn-miRs with their targets for several GO terms.

**Figure 6:**
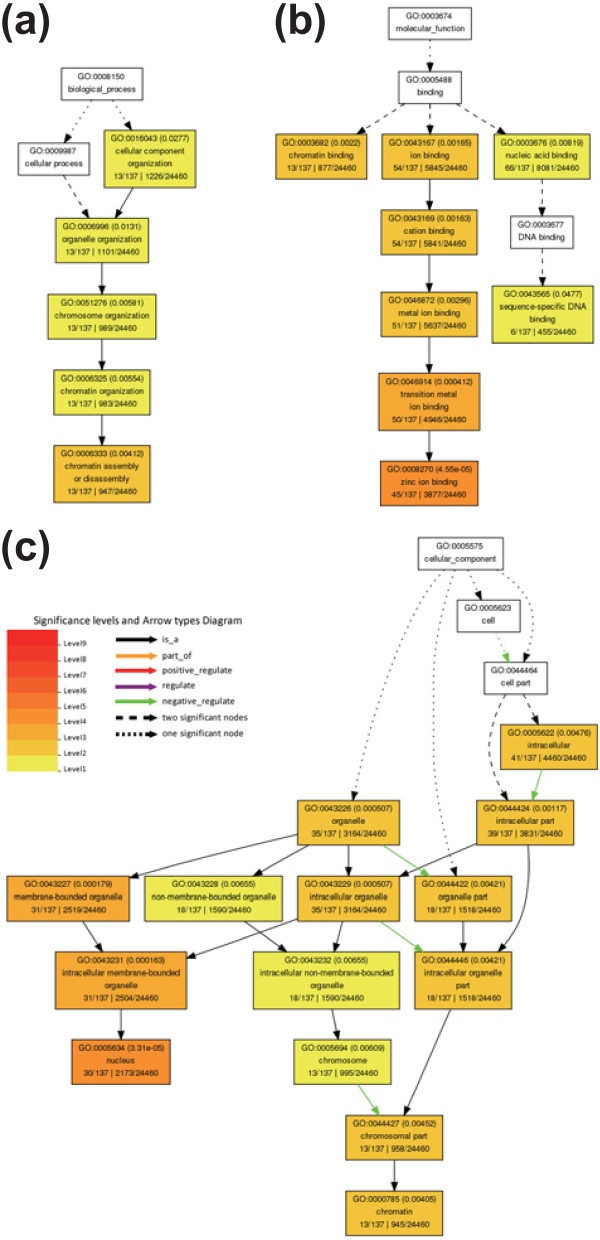
**Gene Ontology term 'enrichment status' for targets of BCP-expressed nov-miRs**. **(a-c) **Targets with GO term 'enrichment status' and 'hierarchy' for (a) biological process, (b) molecular function and (c) cellular component branches. The classification terms and their serial numbers are represented as boxes. For significant terms, the box includes the GO term, adjusted *P*-value (in parentheses), item number mapping the GO term in the query list and background, and total number of items in the query list and background. The color scale shows the *P*-value cutoff levels for each biological process; the more statistically significant, the darker and redder the color.

However, targets of the nov-miRs were more versatile, with very few transcription factors but more transposable elements and functionally unknown transcripts (Additional file 8b) in contrast to the functional terms of targets of kn-miRs (Additional file 8a). Furthermore, the expression of most nov-miRs was negatively associated with that of their targets (Additional file 10b); the expression of only a small proportion positively correlated with that of their targets. Statistical analysis of GO terms demonstrated that terms related to chromatin assembly and disassembly were significant for targets of BCP-expressed nov-miRs (Figure 6), but there were no significant GO terms associated with targets of UNM-and TCP-expressed nov-miRs. This finding suggests that pollen-expressed nov-miRs may have different roles from pollen-expressed kn-miRs in developing pollen.

### miRNA-induced cleavage of predicted targets

Our observation that most of nov-miRs and their targets had negatively correlated expression (Additional file 10b) implies that these nov-miRs have potential cleavage activity. To validate the cleavage events of nov-miRs, we amplified their predicted target genes using rapid amplification of 5' cDNA ends (5' RACE).

**Figure 7:**
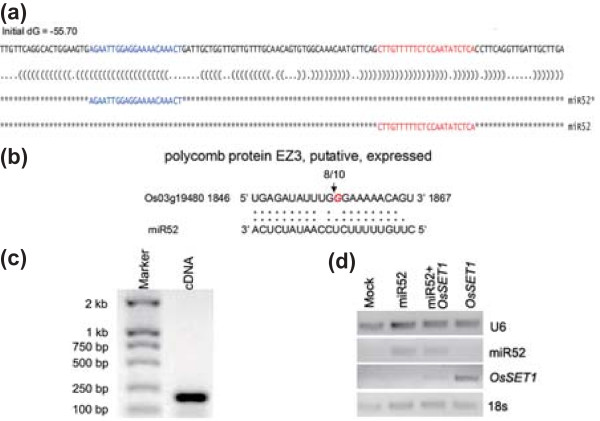
**Target cleavage validation of miRNA52**. **(a) **The miRNA hairpin of miR52 shown in bracket notation format. Mature miRNA is in red and miRNA* in blue. **(b) **Cleavage pattern of *OsSET1 *by miR52. **(c) **Gel image showing 5' RACE reaction to detect the miRNA-directed cleavage. **(d) **Transient co-expression of miR52 and *OsSET1 *in *Nicotiana benthamiana *leaves.

miR52, whose pre-miRNA sequence could form a characteristic fold-back structure and produced the complementary miRNA* sequence, was capable of cleaving its target *Os03g19480 *(encoding polycomb protein EZ3, namely *OsiEZ1*/*OsSET1*; see Discussion; Figure 7). Among ten sequenced clones (Figure 7c), eight were detected to have the target sequence in their coding sequence and were cleaved between nucleotides 10 and 11 relative to the 5' end of the complementary miR52 (Figure 7b). Transient co-expression of miR52 and *OsSET1 *in *Nicotiana benthamiana *leaves showed that the level of *OsSET1 *was reduced by miR52 (Figure 7d).

Although, to date, the canonical cleavage site of almost all of the kn-miRs was between nucleotides 10 and 11, we unexpectedly found that miR56 and miR58 cleaved targets with high frequency (10 of 10 for miR56, 5 of 16 for miR58) between nucleotides 18 and 19 and 5 and 6, respectively (Additional file 11). These cleavage events were validated by transient co-expression in *N. benthamiana *leaves (Additional file 11). Furthermore, cleavage of the non-conserved kn-miR osa-miR820 occurred primarily at the canonical position with a frequency of nine out of ten in a cDNA pool from seedling and inflorescence [31] or seven out of eight in a mixture of cDNA from panicle and embryogenic calli [32] but infrequently (one of ten) between nucleotides 11 and 12 from the complementary 5' end of osa-miR820 [31]. However, we found that osa-miR820-mediated cleavage of *Os03g02010 *was predominantly at nucleotide 7 from the paired 3' end (5 of 14) from the mixed cDNA pool from developmental pollen, and no other degradation fragments were detected with the targeted sequence (data not show). As indicated in a study of *Arabidopsis*, the cleavage frequency and cleavage sites of targets for several conserved kn-miRs differed between pollen and vegetative tissues [9], which suggests that the differences might account for subtle regulatory mechanism.

Recently developed degradome sequencing is a useful and powerful tool to discover new targets for conserved and non-conserved miRNAs and to validate putative targets of predicted nov-miRs [33-37]. We searched degradome data for 3-week-old seedlings and young inflorescences of rice [36,37] and starBase (sRNA target Base) [38] to identify the cleavage products of nov-miRs. From the degradome data, we found eight predicted targets with only one count each for eight different nov-miRs (Additional file 12), and from starBase, three predicted target fragments for three different nov-miRs were identified. Among the eleven nov-miRs, three were expressed only in sporophytic tissues and eight were enriched in developing pollen (two in UNMs, two in TCP) or in two successive stages (two in UNMs and BCP), or in both sporophytic tissues and pollen (two of five examined samples except TCP) (Additional file 12). Because of the extremely low frequency of degradation products of the targets mentioned above, detecting them by 5' RACE is almost impossible; indeed, we detected no products for them.

## Discussion

Using next-generation high-throughout sequencing technology, we have analyzed small RNAs in developing rice pollen from the UNM to TCP stages at the genome-wide level. This analysis revealed dynamic features of small RNA populations in the developing pollen and expression patterns of pollen-expressed miRNAs. We also revealed a set of target genes of pollen-expressed miRNAs and the possible relationship between pollen-expressed miRNAs and their targets by analysis of their expression patterns. These results provide novel insights into molecular regulation mediated by miRNAs of pollen development.

### Dynamic changes in small RNA populations in developing pollen

In plants, transcriptional and post-transcriptional gene silencing is directed by genome-encoded 21-to 24-nucleotide small RNAs. miRNAs of 21 nucleotides and *trans*-acting siRNAs of 21 nucleotides function in post-transcriptional gene silencing by guiding mRNA degradation or translational repression [1,39-41]. siRNAs of 24 nucleotides are implicated in DNA and histone modifications leading to transcriptional gene silencing [40-43], and 24-nucleotide miRNAs involved in DNA methylation were also reported recently [44]. Recent studies have revealed that the size distribution of small RNAs is tissue-specific in maize [45]. Mature pollen of *Arabidopsis *loses most 24-nucleotide siRNAs and gains some 21-nucleotide siRNAs, which target silencing in the pollen [7]. Using high-throughout Solexa sequencing, we obtained more than 5 million high-quality small RNAs from pollen samples at three developmental stages and from sporophytic tissues. The size distribution of small RNAs varied with pollen development. The number of small RNAs of 24 nucleotides, which would be mainly siRNAs, peaked in UNMs and BCP, with more 20-and 21-nucleotide small RNAs (mainly miRNAs) in TCP (Figure 1). This finding suggests that the shift in small RNA populations in the developing pollen may represent a mechanism regulating the gene expression program and finally pollen development. Also, transcriptional regulation would be an important mechanism in the early phase, but post-transcription regulation would be prevalent in the late phase of pollen development in rice.

### Developing pollen has a unique expression pattern of miRNAs

miRNAs are a highly diverse class of small RNAs; hundreds of mature sequences have been registered in miRBase [16-18]. However, almost all of them, if not all, were identified originally from sporophytes. We knew little about the miRNAs in pollen. miR164 and miR171 were first discovered to be expressed in pollen of *Nicotiana *by northern blot analysis [46], and miR164 was further identified in pollen of *Arabidopsis *[47]. Recent studies of *Arabidopsis *revealed the expression of some kn-miRs in mature pollen [11] and the expression of critical components of small RNA pathways in developing pollen [9-11]. Our study revealed 292 kn-miRs from pollen and sporophytic tissue libraries, 202 of which were expressed in developing pollen. Among the 202 pollen-expressed kn-miRs, many (103) were even pollen enriched (see Results for details). These results indicate that most of the kn-miRs originally identified from sporophytes were also expressed or even enriched in developing pollen. In contrast, most kn-miRs detected in *Arabidposis *mature pollen were in low abundance [11], possibly because the mature pollen is in a terminal development status ready for fertilization and needs no more miRNAs for regulation. Therefore, the existence and high expression of kn-miRs in developing rice pollen demonstrates their importance during pollen development.

Impressively, among 20 miRNA families now recognized to be conserved in diverse plant species [48,49], 16 were identified in developing pollen and sporophytic tissues in this study, and 6 (miR156, miR159, miR164, miR166, miR167 and miR396) were accumulated highly throughout all examined samples (clusters 4, 5 and 9 in Figure 2a), which implies that these conserved miRNAs are conserved among species and among sporophytes and pollen, and among developmental stages of pollen. These conserved miRNAs may be integral regulators of both sporophytic and gametophytic development, and some have house-keeping functions in plant cells.

Although most studies focus on the more conserved and nonspecific miRNA families in plants, given the fast evolution of miRNA sequences, several studies have demonstrated the presence of recently evolved plant species-specific miRNAs [50]. Our work revealed 75 nov-miRs in the 6 libraries. Unexpectedly, more than half of these nov-miRs were expressed only in developing rice pollen (clusters 1 to 3 and 6 to 8 in Figure 2b), and many (clusters 6 to 8 in Figure 2b) were detected only in pollen samples at individual developmental stages, which demonstrates the existence of pollen-specific miRNAs at one or more stages, at least in rice. Vascular plants evolved increasingly complex sporophytes with reduced gametophyte complexity and size, and ultimately loss of independence [51], and the gametophyte in flowering plants has been reduced to just a few cells in pollen grains. The high proportion of nov-miRs specific to pollen may be due to the need for this evolved development of the gametophyte.

Furthermore, we found differential expression of miRNAs in developing pollen and sporophytic tissues. Principal component analysis revealed gametophytes are distinct from sporophytes with regard to the expression profiles of miRNAs, with nov-miRs and non-conserved kn-miRs as the major contributors to this distinction (Figure 3). Therefore, miRNA expression features in pollen differ significantly from those in sporophytic tissues, which suggest a unique regulation program in developing pollen. Consistent with animal sperm cells, plant gametophytes function to faithfully maintain selfish genetic information and developmental potential in establishing the subsequent generation of an individual. Several studies of mammalian organisms have demonstrated that miRNAs enriched in sperm cells are crucial for global regulation of the germ cell developmental program and for keeping selfish genetic elements under strict surveillance [52]. Together, these data demonstrate the involvement of miRNA-mediated gene expression regulation in pollen development.

### Roles of miRNAs in pollen development

Post-meiotic pollen development involves a series of fine-tuned, coordinated cellular events associated with unique transcriptomic profiles, along with differential expression of a complex set of transcription factors [5,53], which indicates crucial roles of gene expression regulation in the development. Recent studies have shown the key function of small RNAs, particularly miRNAs and siRNAs, in regulating gene expression in both plant and animal species [1,3,41] and their crucial roles in regulating sperm cell development and maintaining stability of the genetic element of the cell in mammalian organisms [52]. Many predicted targets encode transcription factors, suggesting that plant miRNAs are master regulators. Our present work found BCP expressed more miRNAs, including known and novel miRNAs (152 kn-miRs and 39 nov-miRs) than UNMs (141 kn-miRs and 30 nov-miRs) and TCP (143 kn-miRs and 18 nov-miRs), and previous research showed that BCP expressed the smallest number of transcription factors [5], indicating that miRNAs cooperate with transcription factors to achieve the trait of gene expression during pollen development, with BCP having the smallest number of stage-enriched transcripts during rice pollen development [5].

Furthermore, a growing number of miRNA-target pairs have been confirmed experimentally (reviewed by [3,41]) and play crucial roles in plant development. For example, overexpression of miR159 repressed mRNA levels of *MYB33 *and *MYB65 *and induced male sterility as well as delayed flowering under short days [54,55]; expression of miR167-resistant *ARF6 *leads to arrested ovule development and indehiscent anthers [56]; *NAC1*-miR164 pair mediates auxin signaling in lateral root emergence [57]; *TIR1*, encoding an auxin receptor, and related F-box genes are regulated by miR393 [58]. Our study identified all of these miRNAs and found some of them were enriched in developing pollen (Additional file 1), and the corresponding targets have already been predicted. We also found that hormone-signaling-related genes were overrepresented in a set of targets of pollen-enriched kn-miRs (Additional file 8a). Hormone signaling in pollen plays crucial roles in coordinating pollen development and maturation [59-63]. Numerous studies have revealed crucial roles of PPR proteins and F-box proteins in pollen development and the importance of chromatin remodeling and DNA repair in maintaining genetic element stability [5,64-67]. Genes associated with these functions were also predicted to be targets of pollen-enriched kn-miRs, suggesting their important roles possibly during pollen development. However, the correlation of expression profiles of pollen-enriched kn-miRs with their corresponding targets was significantly different from that of sporophyte-enriched kn-miRs with their targets (Figure 5). It is possible, therefore, that some sporophyte-originated kn-miRs have roles in different mechanisms when expressed preferentially in pollen.

In contrast to kn-miRs, more than half of the identified nov-miRs were expressed only in developing pollen, with several being stage-specific, and their targets were more versatile. Impressively, GO abundance analysis revealed that terms related to chromatin assembly and disassembly were over-represented in the set of targets of BCP-expressed nov-miRs (Figure 6). Chromatin assembly and disassembly are fundamentally important processes that are tightly linked to DNA replication and transcription, thus ensuring that cells faithfully duplicate chromosomes. This process is essential for developing pollen to coordinate germ cell development and strict maintenance of genome integrity and stability.

Several mechanisms involve chromatin assembly and disassembly, including chromatin remodeling and modification of core histones or cytosines in CpG-rich regions of the genome. Recent studies revealed histone remodeling-related genes targeted by miRNAs expressed in mature pollen of *Arabidopsis*, such as miR778, which targets transcripts of two histone methyltransferase genes, *SUVH5 *and *SUVH6 *[10,68]. A gene encoding the SET domain protein OsSET1/OsiEZ1 was verified to be the target of BCP-specific miR52 and was negatively regulated by this miRNA (Figure 7). SET-domain proteins, chiefly responsible for lysine methylation of histone H3, such as SDG4/ASHR3, are involved in pollen development [69]. Furthermore, our 5' RACE analysis, in combination with several previous reports [31,32,44], confirmed that osa-miR820, which is enriched in pollen, targeted a DNA cytosine methyltransferase gene (*Os03g02010*). Our study also revealed that osa-miR827, which is highly expressed in pollen, targeted *Os04g11510*, which encodes a methyl-CpG binding domain protein (Additional file 11). Both of these proteins are important to regulate the methylation of a genome [70-73]. The epigenetic modification of histones and cytosines in the genome of mammalian germ lines is essential for sperm cell development and plays roles in parental imprinting [74,75]. These results indicate that epigenetic regulation of chromatin assembly and disassembly may be an important mechanism underlying pollen development and may involve parental imprinting. Further function studies of genes are needed to clarify these roles.

## Conclusions

Using Solexa high-throughout sequencing technology, we sequenced the small RNA population of rice pollen at three sequential developmental stages from microspores to tricellular pollen, with sporophytic tissues-roots, leaves, and callus cells-as controls. We obtained millions of high-quality readouts from each sample and identified 292 kn-miRs and 75 nov-miRs. The miRNA composition and expression pattern of developing pollen were obviously different from those of sporophytes, with more nov-miRs enriched/specifically expressed in pollen while more kn-miRs were enriched/specifically expressed in sporophytes. Principal component analysis revealed that pollen could be differentiated from sporophytes with regard to miRNA expression profiles, with novel and non-conserved known miRNAs the main contributors to this. Furthermore, 1,068 targets were predicted for 292 known miRNAs; although no obvious differences were found by GO abundance analysis of those targets between pollen-enriched and sporophyte-enriched kn-miRs, correlation of expression profiles of pollen-enriched kn-miRs with their targets significantly differs from that of sporophyte-enriched kn-miRs with their corresponding targets in terms of transcription, hormone signaling and chromatin remodeling. We identified 285 targets for the 75 nov-miRs, which appeared to be negatively regulated. GO terms for chromatin assembly and disassembly were statistically significantly associated with the targets of BCP-expressed nov-miRs, implying that BCP would be the key point of miRNA regulation. Our data reveal for the first time comprehensive and dynamic features of miRNAs in developing pollen.

## Materials and methods

### Plant materials

Rice cultivar Zhonghua 10 (*O. sativa *L. ssp. *japonica*) was planted in a climate chamber under a 12-hour light/12-hour dark cycle at 28°C for 2 weeks, then the roots and leaves were collected. One-month-old callus cells were generated from rice embryos on N6 solid medium containing 2, 4-Dichlorophenoxyacetic acid (2 mg/l) in the dark at 25°C. Pollen at UNM, BCP and TCP stages was obtained as described previously [5].

### Small RNA extraction

Total RNA was extracted from sporophytic tissues and pollen at each developmental stage by use of RNAplant reagents (Tiangen Biotech, Beijing, China) according to the manufacturer's instructions. Total RNA was resolved on a denatured 15% polyacrylamide gel, then the fraction from 18 to 30 nucleotides was collected.

### Solexa sequencing

The sequencing was performed as described [76]. In brief, purified small RNA molecules were ligated to a pair of Solexa adaptors at the 5' and 3' ends and amplified by use of adaptor primers for 15 cycles to produce sequencing libraries. PCR products were purified and small RNA libraries were sequenced by use of Solexa.

### Computational analysis of sequencing data

Small RNA reads were produced by use of the Illumina 1 G Genome Analyzer (Illumina, San Diego, CA, USA). Our raw data have been deposited in the European Nucleotide Archive by ArrayExpress [77] [ArrayExpress: E-MTAB-689]. After filtering the low quality reads, and trimming adaptor sequences by a modified dynamic programming algorithm [78], we collected short RNAs ranging from 18 to 30 nucleotides and drew size distributions. Sequences of 18 or more nucleotides were mapped to the rice genome (TIGR version 5.0) by SOAP v1.11 [15]. Sequences perfectly matching the genome along their entire length were considered for subsequent analyses. Sequences matching known rice rRNAs, tRNAs, snRNAs and snoRNAs in the Rfam RNA family database [79-81] and NCBI GenBank database [82] were discarded. With the available annotation of repeat sequences in the rice genome (TIGR *Oryza *Repeat Database version 3.3) and use of a parallel overlap finding algorithm, small RNAs positioned at repeat loci were identified and annotated as repeat-associated small RNAs. Degraded species of mRNAs can also be sampled out by Solexa technology, and sequences overlapping with gene regions were excluded.

Sequences mapped to miRNA precursors found by BLAST search of the miRBase miRNA Database (v16.0) [16-18] were identified as kn-miRs, with a manual check to delete the false miRNAs. miRNA precursors have a characteristic fold-back structure, which can be used to predict nov-miRs. By folding the flanking genome sequence of small RNAs, then analyzing its structural features, we could identify novel miRNA candidates by use of MIREAP [27]. Good candidate sequences were then submitted to a target prediction web server containing plant miRNAs [29].

### Principal component analysis of miRNAs

The normalized reads (TPM) of all miRNAs detected and predicted in this study were transformed with use of the log2 scale and analyzed by SIMCA-P+ 12.0 with PCA-X (Ctr scaling) following the PLS-DA model to obtain the principal components. miRNAs with a score contribution more than the absolute value of 2 were illustrated by comparing different samples or tissue types.

### Quantitative RT-PCR of miRNAs

Total RNA was isolated from pollen at each developmental stage and from sporophytic tissues. To prepare small RNA, 0.5 M NaCl and 5% PEG8000 were used to precipitate and remove high-molecular-weight RNA. Small RNA in the resulting supernatant was pelleted with a 1/10 volume of 3 M sodium acetate and 3 volumes of ethanol [36]. The concentration of small RNAs was determined by use of the Beckman Coulter DU730 Nucleic Acid/Protein Analyzer (Fullerton, CA, USA). cDNA was synthesized in a 10-μl reverse transcription (RT) reaction using of 100 ng purified small RNA, 1 U ReverTra Ace reverse transcriptase (Toyobo, Osaka, Japan), 2 μl of 5 × RT buffer, 1 mM dNTPs and 2.4 U Ribnuclease inhibitor (TAKARA SHIZU CO., LTD, Tokyo, Japan), and 50 nM miRNA-specific stem-loop primers (Additional file 13) designed according to Chen *et al*. [83]. U6 snRNA was chosen as a reference and was reverse-transcribed with reverse primer OsU6R [84,85]. A pulsed RT procedure was used to increase the reaction specification [86]. In brief, the RT reaction mixture was incubated at 16°C for 30 minutes, then 60 cycles for 30 s at 30°C, 30 s at 42°C, and 1 s at 50°C, finally at 95°C for 5 minutes. No RNA or RT primers or RT controls were set at the same time. SYBR^® ^Green Realtime PCR Master Mix (Toyobo) was used to detect miRNA expression by a Stratagene Max 3000p Detection System (La Jolla, CA, USA). Briefly, cDNAs were diluted 5 times and 1 μl diluted product was used as a template in a 10-μl PCR reaction, which contained 5 μl 2 × SYBR Green Realtime PCR Master Mix and 0.25 μM of an miRNA-specific forward primer and universal reverse primer. The quantitative PCR was conducted in duplicate for 90 s at 95°C, then 40 cycles of 15 s at 95°C and 10 s at 60°C. For each PCR, dissociation curve analysis was carried out to discriminate the specific products from the primer dimers. The fold changes of miRNA in different samples were calculated by the 2^Δ ΔCt ^method as described [5].

### 5' RACE of miRNA cleavage

Total RNA (1 μg) from pollen of equally mixed UNMs, BCP and TCP was used to synthesize 5'-RACE-ready cDNAs with N-15 random primer mix and BD Smart RACE cDNA Amplification Kit (Clontech, Palo Alto, CA, USA) according to the manufacturer's instruction. The first round of PCR involved 10 × UPM, outer gene-specific primers and Advantage 2 Polymerase Mix (Clontech). The product was diluted 50 times and then used as a template for the second round of PCR, which involved NUP and outer/inner gene-specific primers. Amplicons were separated on the gel, cloned into pMD 19-T vector (Takara) and sequenced. The outer and inner gene-specific primers were listed in Additional file 13.

### Transient co-expression of miRNAs and their targets in *N. benthamiana *leaves

Rice genomic fragments forming fold-back structure for precursors of miR52, miR56, miR58 and osa-miR827a carrying the miRNA and/or miRNA* duplex were amplified with the primers listed in Additional file 13 and cloned into a binary vector (pTCK303) driven by the maize ubiquitin promoter. Their corresponding target fragments obtained with primers listed in Additional file 13 were inserted into the same vector. The plasmids carrying precursors of miRNAs and targets were infiltrated with separate or mixed cells as described [37,87]. The total RNA of infiltrated leaves was isolated after 4-day growth and used to synthesize the first cDNA as templates of semi-quantitative real-time PCR as mentioned above.

## Abbreviations

AGO: Argonaute; BCP: bicellular pollen; DCL: Dicer-like protein; GO: Gene Ontology; kn-miR: known miRNA; miRNA: microRNA; nov-miR: novel miRNA; PPR: pentatricopeptide repeat; RACE: rapid amplification of cDNA ends; RT: reverse transcription; siRNA: small interfering RNA; snRNA: small nuclear RNA; snoRNA: small nucleolar RNA; TCP: tricellular pollen; TPM: transcripts per million; UNM: uninucleate microspore.

## Authors' contributions

TW designed the experiments and wrote the paper. LQW performed most of the experiments and data analysis, and wrote the draft of the paper. LFY carried out the real-time quantification RT-PCR, 5' RACE of miRNA cleavage and principal component analysis of miRNAs. All authors read and approved the final manuscript.

## Supplementary Material

Additional file 1 **All identified known miRNAs**. Click here for file

Additional file 2 **Small RNA sequences perfectly matching known miRNA hairpins**. Click here for file

Additional file 3 **Small RNA sequences perfectly matching predicted novel miRNA hairpins**. Click here for file

Additional file 4 **All predicted novel miRNAs**. Click here for file

Additional file 5 **Clustering of all known miRNAs by k-means support**. The six points from the left to the right in the x-axis represent uninucleate microspores (UNMs), bicellular pollen (BCP), tricellular pollen (TCP), callus cell, root and leaf, respectively; the y-axis represents the log2 value of transcripts per million. Click here for file

Additional file 6 **Clustering of all novel miRNAs by k-means support**. The six points from the left to the right in the x-axis represent UNM, BCP, TCP, callus cell, root and leaf, respectively; the y-axis represents the log2 value of transcripts per million. Click here for file

Additional file 7 **Validation of miRNAs by stem-loop real-time quantitative RT-PCR**. Click here for file

Additional file 8 **Targets of all known miRNAs and novel miRNAs**. **(a) **Kn-miRs; **(b) **nov-miRs. Click here for file

Additional file 9 **Gene ontology term 'enrichment status' for targets of pollen-enriched and sporophyte-enriched known miRNAs**. Click here for file

Additional file 10 **Expression profiles of known miRNAs and novel miRNAs and their targets**. **(a) **Kn-miRs and targets; **(b) **nov-miRs and targets. Click here for file

Additional file 11 **Targets cleavage by miRNAs**. **(a) **The predicted fold-back structures of miR56 and miR58. Mature miRNA is in red. **(b) **Cleavage pattern of targets by corresponding novel miRNAs. **(c) **Gel image showing 5' RACE reaction to detect the miRNA-directed cleavage. **(d) **Transient co-expression of miRNAs and their predicted targets in *N. benthamiana *leaves. As a positive known miRNA, the co-expression of osa-miR827a and its target *LOC_Os04g11510 *is also shown. Click here for file

Additional file 12 **Predicted target fragments of novel miRNAs from degradome data**. Click here for file

Additional file 13 **Primers used in this study**. Click here for file
