# Supplementary material for: Deep sequencing on genome-wide scale reveals the unique composition and expression patterns of microRNAs in developing pollen of Oryza sativa
Source: Genome Biol. 2011 Jun 16;12(6):R53. doi: 10.1186/gb-2011-12-6-r53 (PMC3218841; doi:10.1186/gb-2011-12-6-r53)
Supplement: Additional file 9 — Gene ontology term 'enrichment status' for targets of pollen-enriched and sporophyte-enriched known miRNAs. [file gb-2011-12-6-r53-S9.PPT]

## Slide 1
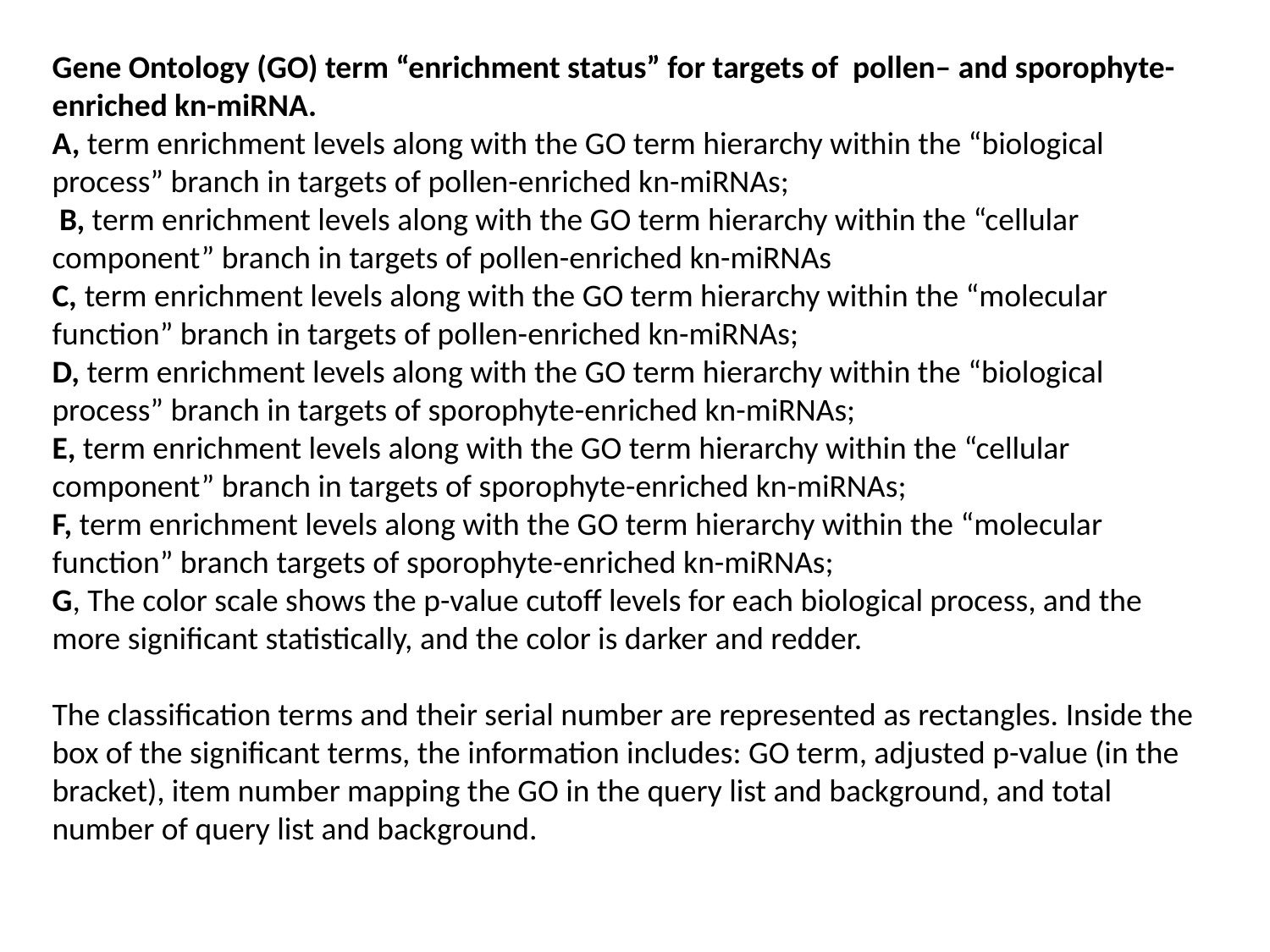

Gene Ontology (GO) term “enrichment status” for targets of pollen– and sporophyte-enriched kn-miRNA.
A, term enrichment levels along with the GO term hierarchy within the “biological process” branch in targets of pollen-enriched kn-miRNAs;
 B, term enrichment levels along with the GO term hierarchy within the “cellular component” branch in targets of pollen-enriched kn-miRNAs
C, term enrichment levels along with the GO term hierarchy within the “molecular function” branch in targets of pollen-enriched kn-miRNAs;
D, term enrichment levels along with the GO term hierarchy within the “biological process” branch in targets of sporophyte-enriched kn-miRNAs;
E, term enrichment levels along with the GO term hierarchy within the “cellular component” branch in targets of sporophyte-enriched kn-miRNAs;
F, term enrichment levels along with the GO term hierarchy within the “molecular function” branch targets of sporophyte-enriched kn-miRNAs;
G, The color scale shows the p-value cutoff levels for each biological process, and the more significant statistically, and the color is darker and redder.
The classification terms and their serial number are represented as rectangles. Inside the box of the significant terms, the information includes: GO term, adjusted p-value (in the bracket), item number mapping the GO in the query list and background, and total number of query list and background.

## Slide 2
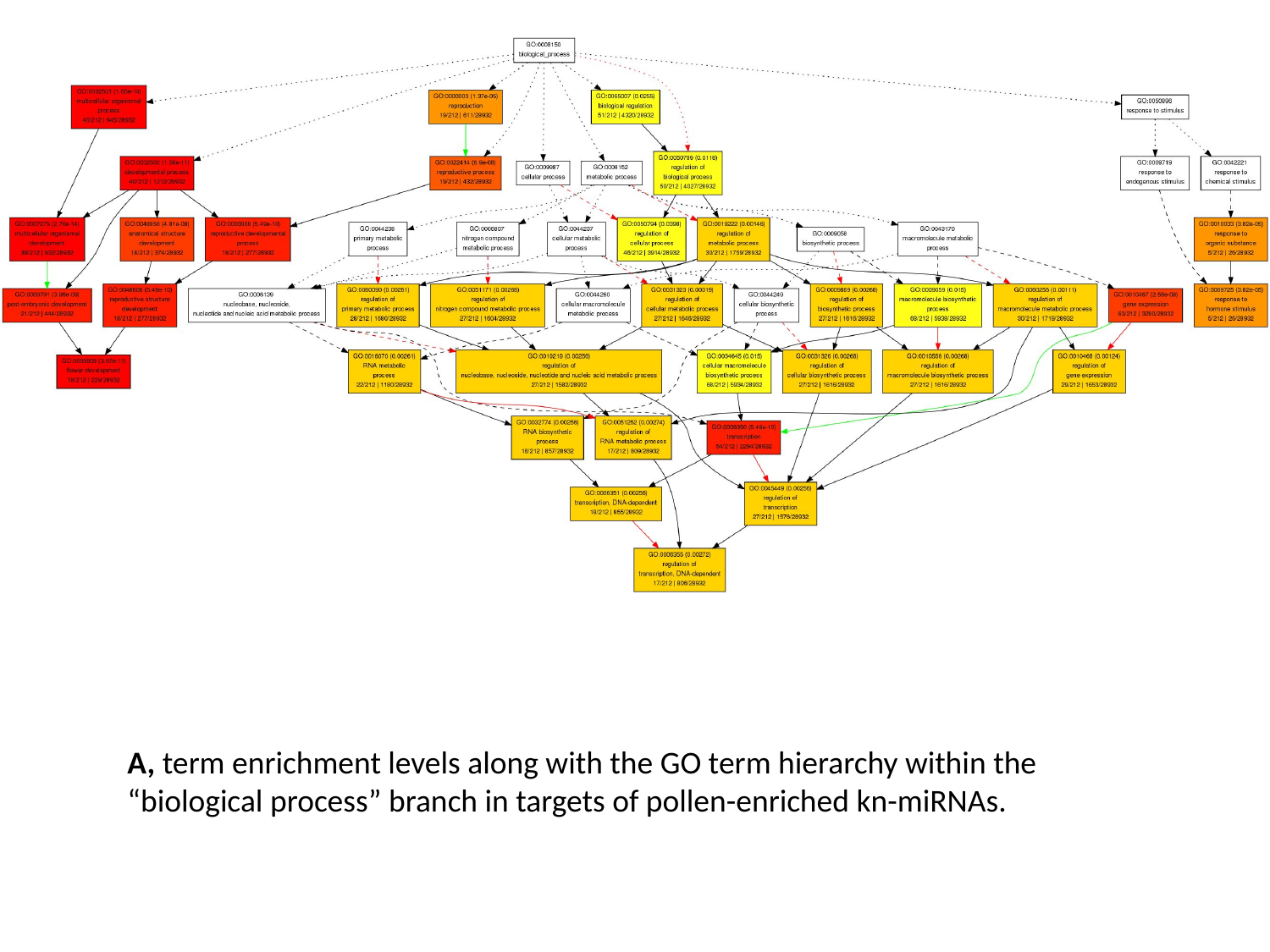

A, term enrichment levels along with the GO term hierarchy within the “biological process” branch in targets of pollen-enriched kn-miRNAs.

## Slide 3
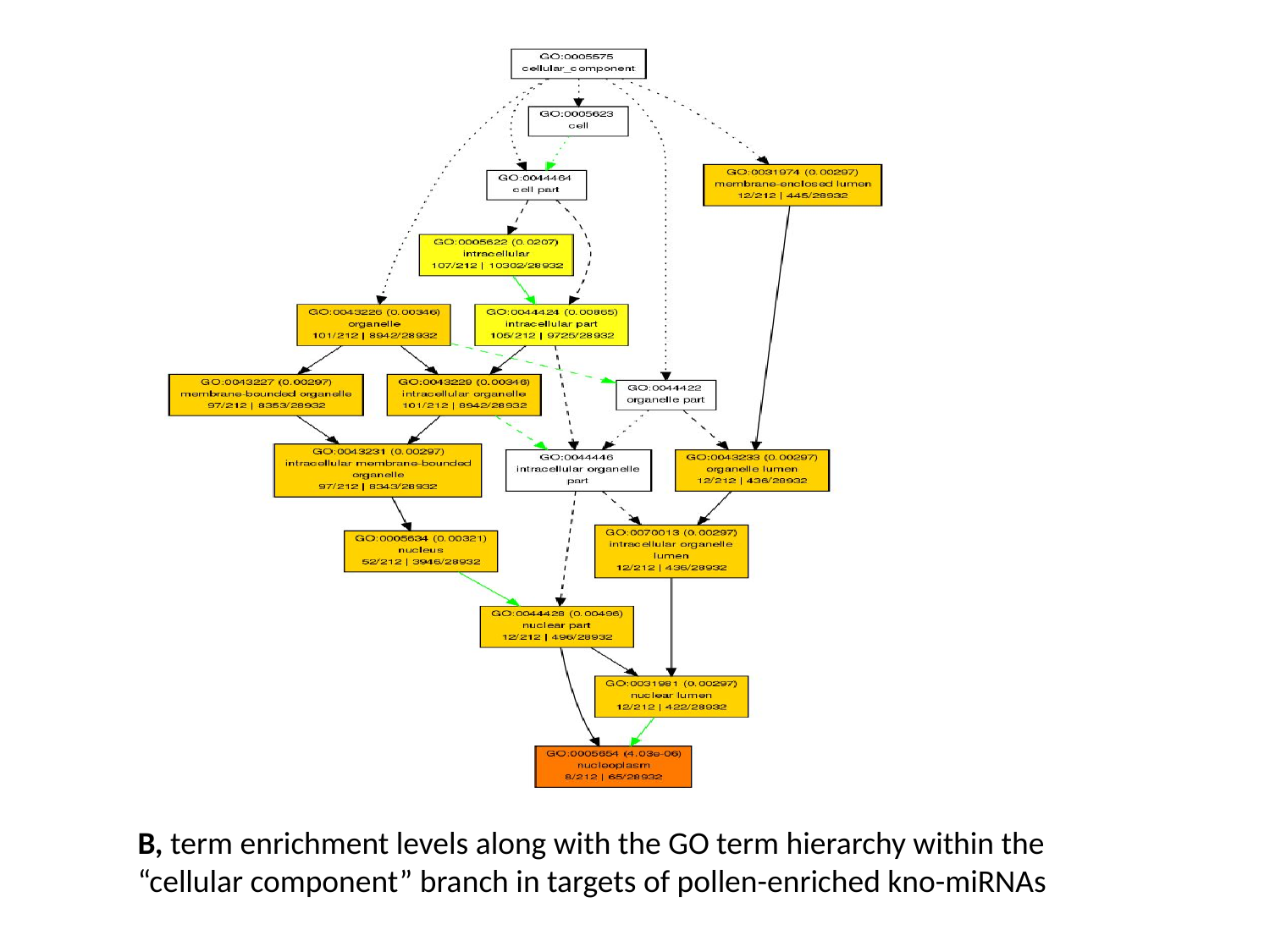

B, term enrichment levels along with the GO term hierarchy within the “cellular component” branch in targets of pollen-enriched kno-miRNAs

## Slide 4
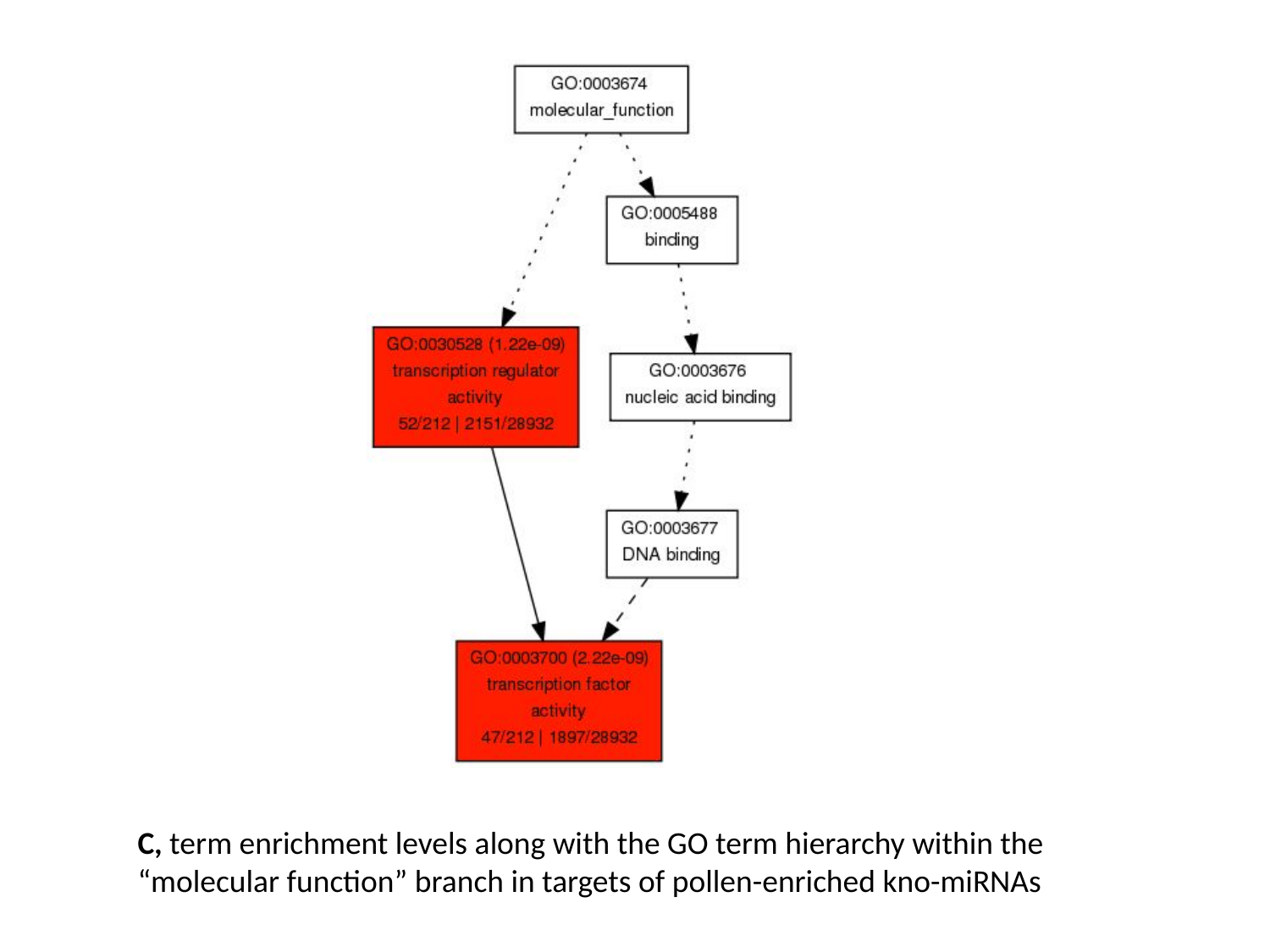

C, term enrichment levels along with the GO term hierarchy within the “molecular function” branch in targets of pollen-enriched kno-miRNAs

## Slide 5
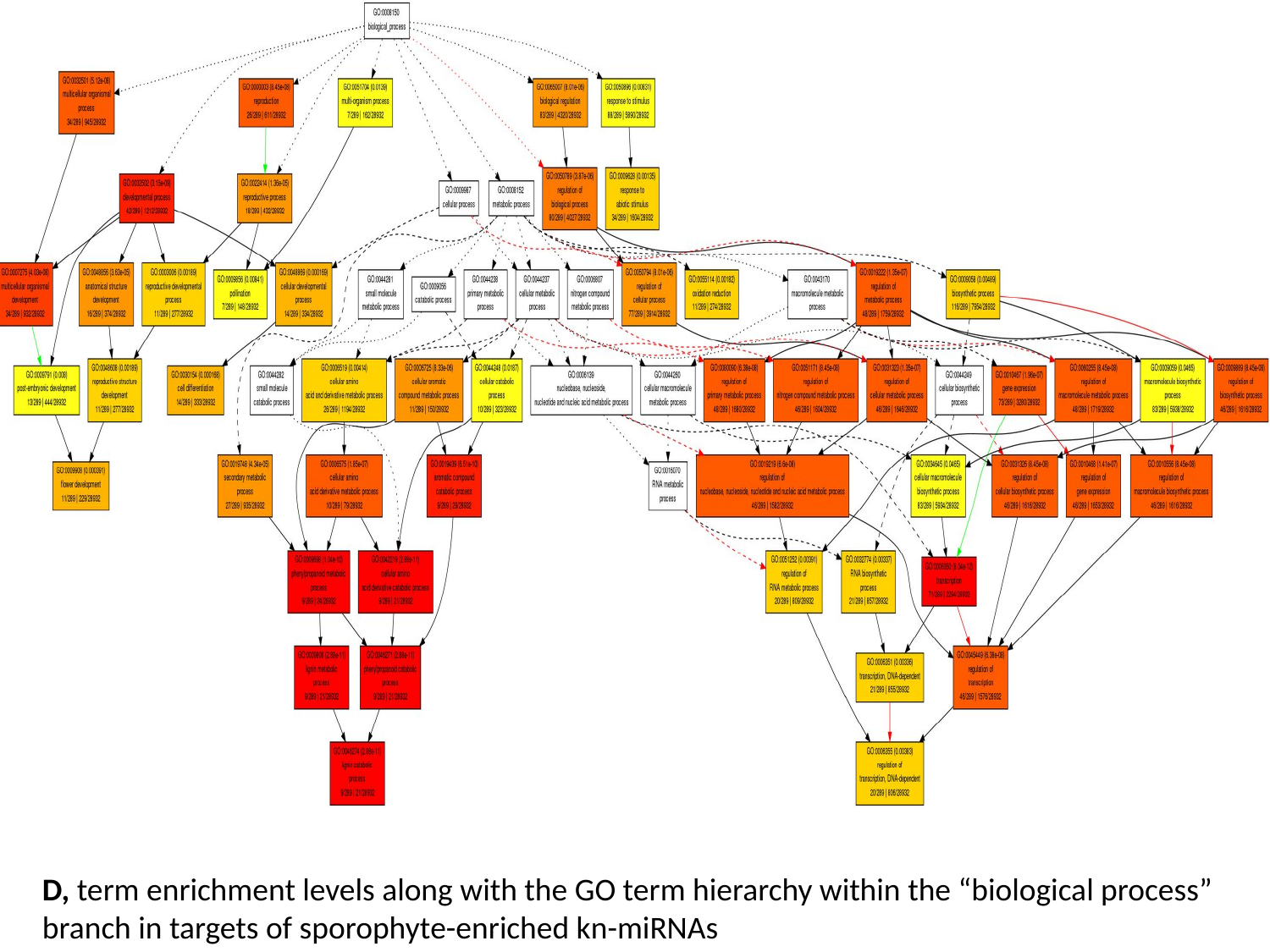

D, term enrichment levels along with the GO term hierarchy within the “biological process” branch in targets of sporophyte-enriched kn-miRNAs

## Slide 6
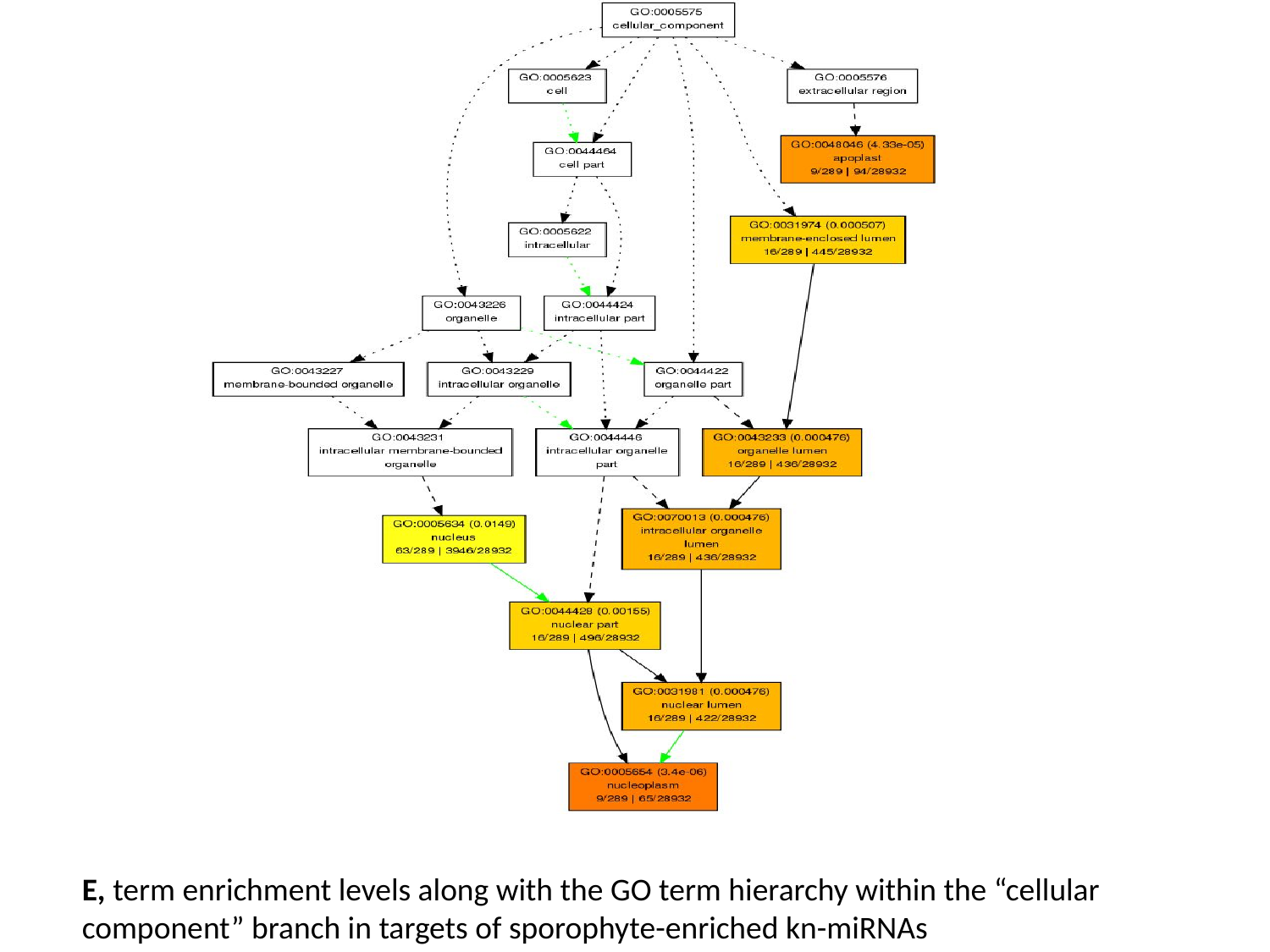

E, term enrichment levels along with the GO term hierarchy within the “cellular component” branch in targets of sporophyte-enriched kn-miRNAs

## Slide 7
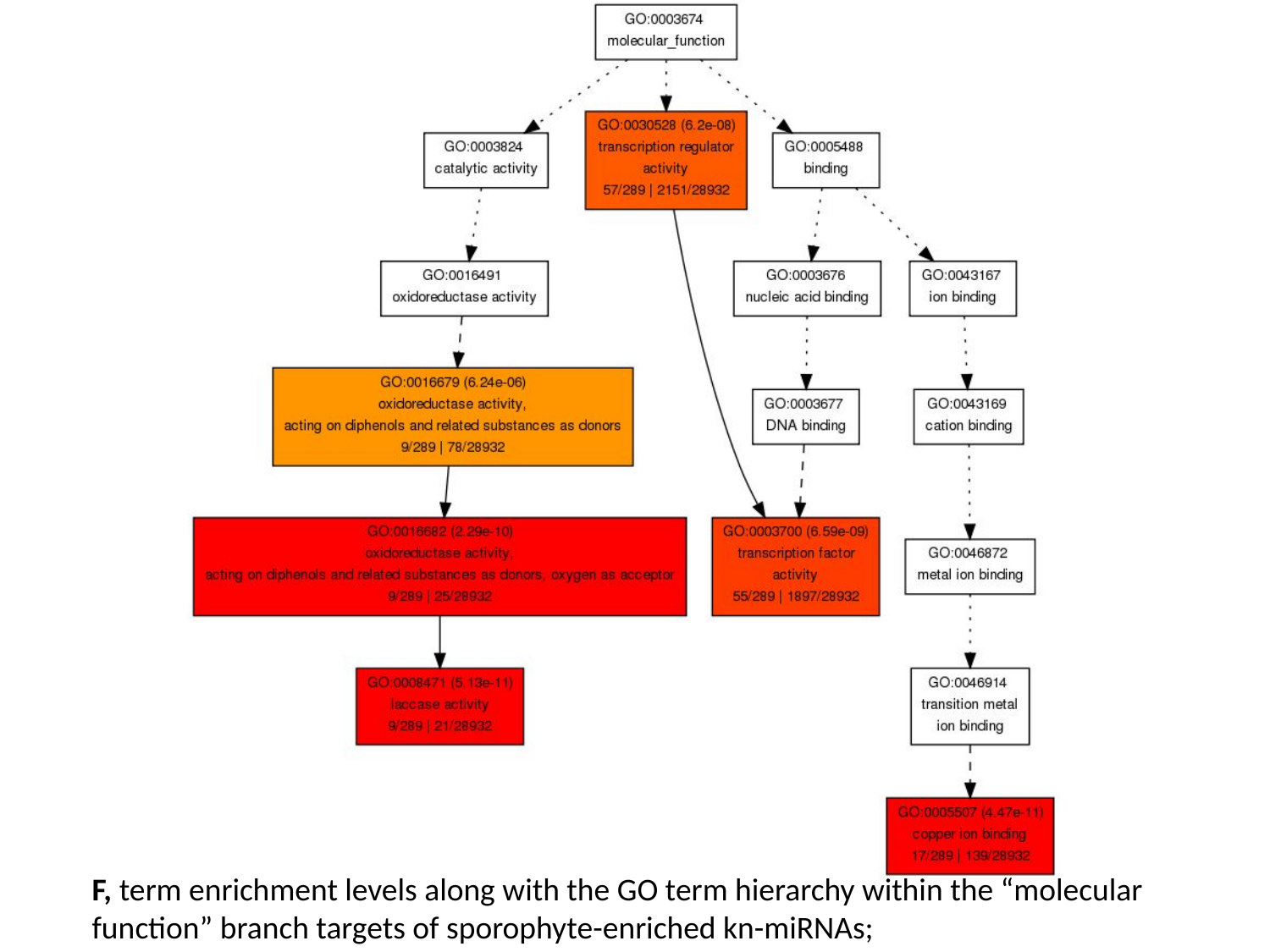

F, term enrichment levels along with the GO term hierarchy within the “molecular function” branch targets of sporophyte-enriched kn-miRNAs;

## Slide 8
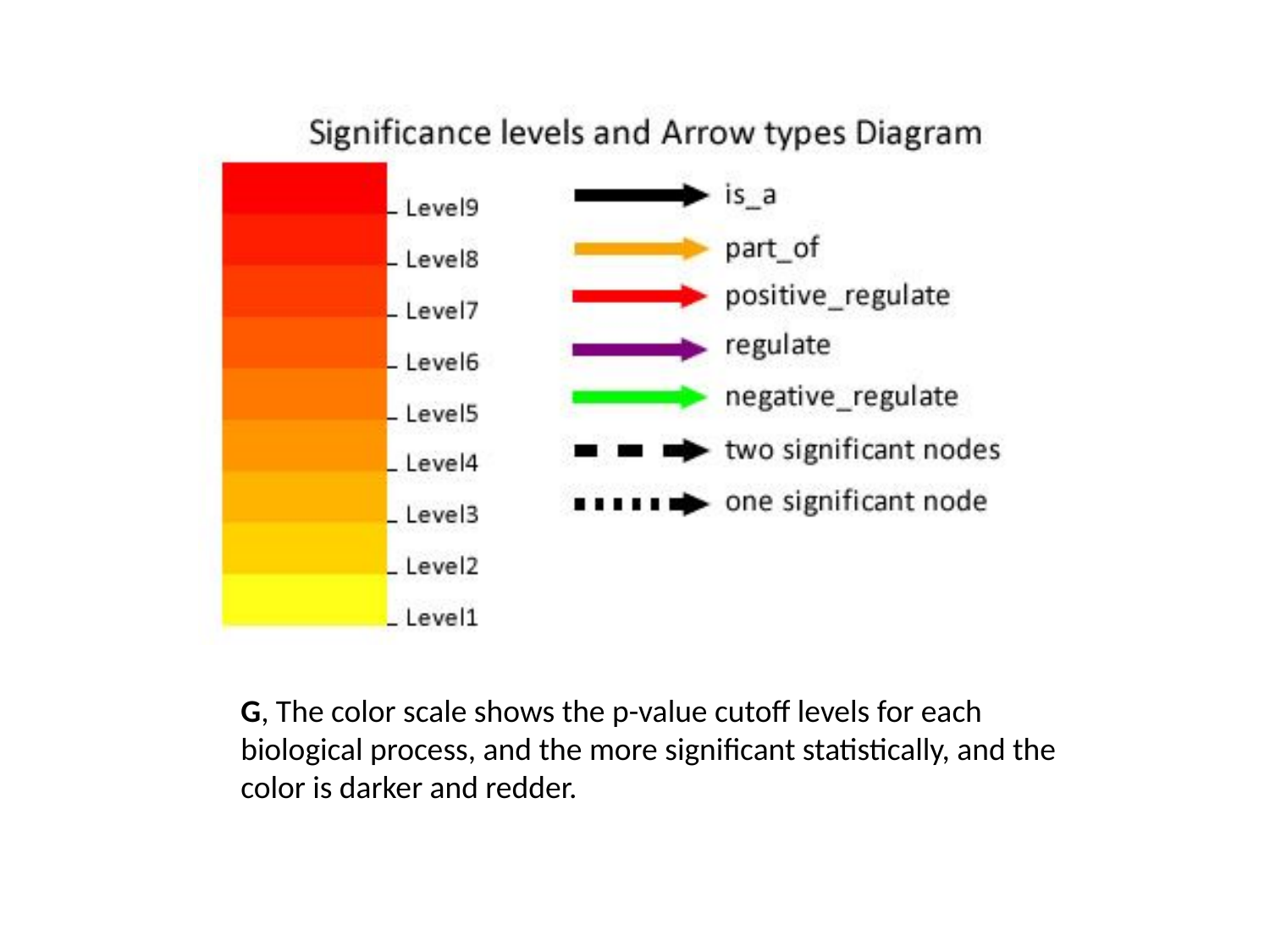

G, The color scale shows the p-value cutoff levels for each biological process, and the more significant statistically, and the color is darker and redder.
